# Triphlorethol A, a Dietary Polyphenol from Seaweed, Decreases Sleep Latency and Increases Non-Rapid Eye Movement Sleep in Mice

**DOI:** 10.3390/md16050139

**Published:** 2018-04-24

**Authors:** Minseok Yoon, Suengmok Cho

**Affiliations:** Division of Functional Food Research, Korea Food Research Institute, Jeollabuk-do 55365, Korea; msyoon@kfri.re.kr

**Keywords:** triphlorethol A, phlorotannins, marine polyphenols, sleep, EEG, hypnotic

## Abstract

In our previous studies, we have demonstrated that marine polyphenol phlorotannins promote sleep through the benzodiazepine site of the gamma-aminobutyric acid type A (GABA_A_) receptors. In this follow-up study, the sleep-promoting effects of triphlorethol A, one of the major phlorotannin constituents, were investigated. The effect of triphlorethol A on sleep-wake architecture and profiles was evaluated based on electroencephalogram and electromyogram data from C57BL/6N mice and compared with the well-known hypnotic drug zolpidem. Oral administration of triphlorethol A (5, 10, 25, and 50 mg/kg) dose-dependently decreased sleep latency and increased sleep duration during pentobarbital-induced sleep in imprinting control region mice. Triphlorethol A (50 mg/kg) significantly decreased sleep latency and increased the amount of non-rapid eye movement sleep (NREMS) in C57BL/6N mice, without affecting rapid eye movement sleep (REMS). There was no significant difference between the effects of triphlorethol A at 50 mg/kg and zolpidem at 10 mg/kg. Triphlorethol A had no effect on delta activity (0.5–4 Hz) of NREMS, whereas zolpidem significantly decreased it. These results not only support the sleep-promoting effects of marine polyphenol phlorotannins, but also suggest that the marine polyphenol compound triphlorethol A is a promising structure for developing novel sedative hypnotics.

## 1. Introduction

Sleep is vital for maintaining health and well-being [1]; the quantity and quality of sleep greatly contributes to physical and cognitive performance, the immune system, stable mood, productivity, and quality of life [2]. Recently, insomnia has become a widespread health issue and has emerged as a prevalent problem in modern society [3,4]. Due to an increase in the number of adults with sleep problems, functional foods or nutritional supplements for sleep improvement are becoming popular as an alternative to prescription sleep medications for treating insomnia or improving sleep quality [5].

For the last two decades, the sedative-hypnotic effects of medicinal plants and their constituents have been extensively investigated [6]. In particular, polyphenols have been considered as major sedative-hypnotic phytochemicals. Polyphenols have long been investigated for their properties as ligands of GABA_A_-benzodiazepine (BZD) receptors that are the major target for sedative-hypnotic agents [7]. In addition, it is generally accepted that polyphenol metabolites can enter the brain through the blood-brain barrier [8,9]. Until now, studies on the sedative-hypnotic effects of polyphenols have been restricted to terrestrial plants, whereas phlorotannins, marine plant polyphenols, have not been recognized as a potential source of natural sedative-hypnotic agents.

In our previous studies, we demonstrated for the first time that brown seaweed extract and its phlorotannin-rich preparation promote sleep in mice via the GABA_A_-BZD receptors [10,11,12]. Phlorotannins, oligomers, and polymers of phloroglucinol, are structurally different from terrestrial plant polyphenols based on gallic acids or flavones [13,14]. They have a high structural diversity, and so far about 150 phlorotannin constituents have been identified [15,16]. However, the sleep-promoting effects of individual phlorotannin compounds are yet to be investigated. In this follow-up study, we investigated the effects of triphlorethol A, one of the major phlorotannin constituents, on sleep in C57BL/6N mice. Analyses of sleep architecture and profiles in mice were performed based on electroencephalogram (EEG) and electromyogram (EMG) measurements.

## 2. Results

### 2.1. Effects of Triphlorethol A on Pentobarbital-Induced Sleep in Imprinting Control Region (ICR) Mice

**Figure 1:**
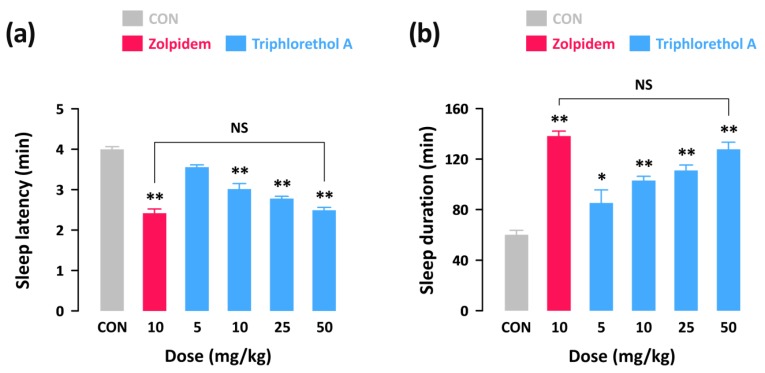
Effects of triphlorethol A and zolpidem on sleep latency (**a**) and sleep duration (**b**) in pentobarbital-treated imprinting control region (ICR) mice. Drugs were administered (p.o.) to mice 45 min before pentobarbital injection (45 mg/kg, i.p.). Each column represents mean ± SEM (*n* = 10). * *p* < 0.05, ** *p* < 0.01, denote cases which were significantly different when compared to the control (CON) group (Dunnett’s test).

In order to investigate whether triphlorethol A produces sedative-hypnotic effects in animals, we first performed the pentobarbital-induced sleep test in ICR mice. With a hypnotic dose of pentobarbital (45 mg/kg, i.p.), sleep latency and duration in the control group were 4.0 ± 0.1 and 60.1 ± 3.5 min, respectively (Figure 1). As expected, a well-known hypnotic drug, zolpidem (10 mg/kg, p.o.) significantly decreased sleep latency (Figure 1a) and increased sleep duration (Figure 1b) as compared to the control group. Triphlorethol A also produced a dose-dependent decrease in sleep latency and increase in sleep duration. In particular, administration of 50 mg/kg of triphlorethol A was found to prolong sleep duration up to 127.7 ± 5.6 min, which is similar to the level of zolpidem at 10 mg/kg (138.2 ± 3.8 min). This result suggests that, similar to zolpidem, triphlorethol A can act as a sedative-hypnotic agent to accelerate pentobarbital-induced sleep in mice.

### 2.2. Effects of Triphlorethol A on Sleep Latency and the Amounts of Rapid Eye Movement Sleep (REMS) and Non-REMS (NREMS) in C57BL/6N Mice

**Figure 2:**
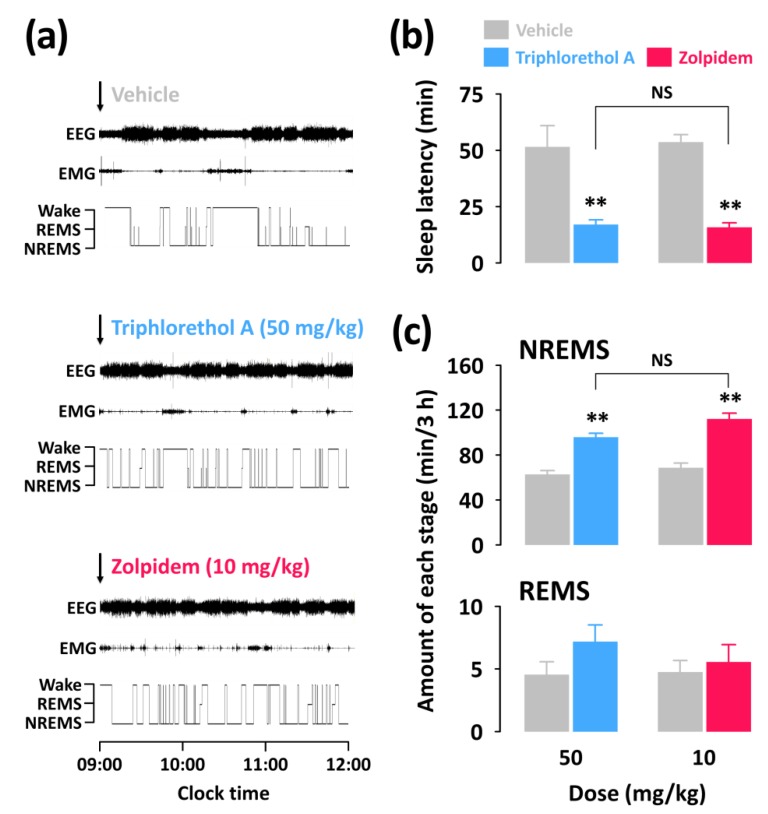
Effect of triphlorethol A and zolpidem on sleep-wake profiles in C57BL/6N mice. (**a**) Representative electroencephalogram (EEG) and electromyogram (EMG) signals, and corresponding hypnograms in a mouse treated with triphlorethol A or zolpidem. (**b**) Effects of triphlorethol A and zolpidem on sleep latency. (**c**) Amounts of rapid eye movement sleep (REMS) and non-REMS (NREMS) during the 3 h period after administration of triphlorethol A or zolpidem. Grey bars indicate the baseline day (vehicle). Each value represents the mean ± SEM of each group (*n* = 7–8). ** *p* < 0.01, denotes significantly different cases from the vehicle (unpaired Student’s *t*-test). Wake denotes wakefulness and NS denotes no significance.

To better understand the sedative-hypnotic effects of triphlorethol A, we performed analyses of sleep architecture and sleep profiles in C57BL/6N mice based on EEG and EMG recordings. For analysis of sleep profiles, we chose a dosage of 50 mg/kg of triphlorethol A and its effects were compared with the reference hypnotic drug zolpidem at 10 mg/kg. In the pentobarbital-induced sleep test, there was no significant difference between triphlorethol A at 50 mg/kg and zolpidem at 10 mg/kg. Figure 2a presents examples of EEG/EMG signals and corresponding hypnograms from a single mouse during the first 3 h following vehicle, triphlorethol A, or zolpidem administration.

The values of sleep latency for triphlorethol A (50 mg/kg) and zolpidem (10 mg/kg) were 16.9 ± 2.1 and 15.8 ± 2.0 min, respectively (Figure 2b). Both triphlorethol A and zolpidem showed significantly (*p* < 0.01) shorter sleep latency compared to each vehicle. This result indicates that triphlorethol A accelerates the induction of NREMS in mice similarly to the hypnotic drug zolpidem. We calculated the amounts of NREMS and REMS during the first 3 h immediately after the administration of triphlorethol A and zolpidem (Figure 2c). As expected, zolpidem increased the amount of NREMS by 3.4-fold (*p* < 0.01) compared to the vehicle. Administration of triphlorethol A was also found to significantly increase NREMS; by 3.0-fold (*p* < 0.01). There was no significant difference between triphlorethol A and zolpidem in terms of the amount of NREMS. Neither triphlorethol A nor zolpidem had a significant effect on the amount of REMS (Figure 2c).

### 2.3. Effects of Triphlorethol A on Time-Course Changes of NREMS, REMS, and Wake in C57BL/6N Mice

**Figure 3:**
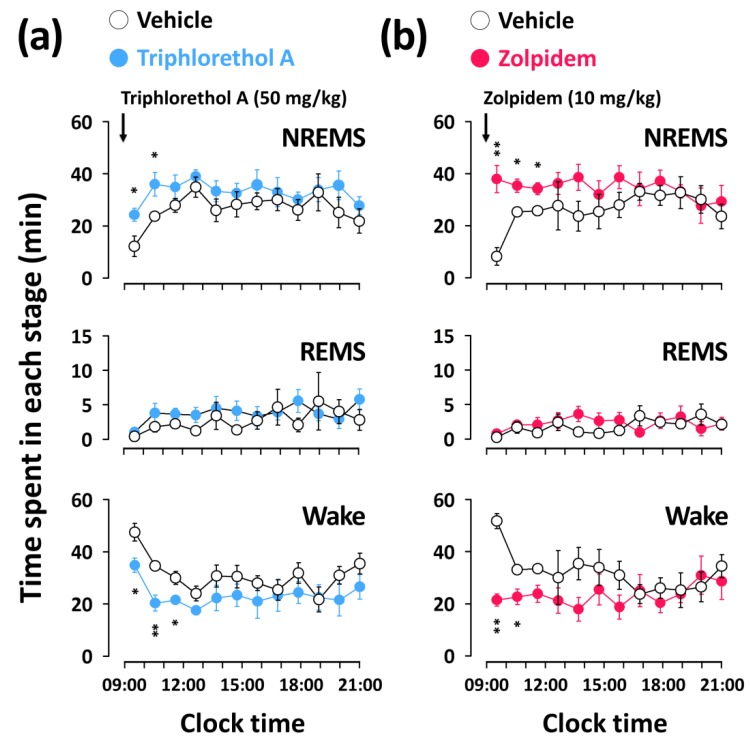
Effects of triphlorethol A (**a**) and zolpidem (**b**) on time-course changes in NREMS, REMS, and wake for 12 h in C57BL/6N mice. Open and filled circles indicate the baseline day (vehicle) and experimental day (triphlorethol A or zolpidem), respectively. Each circle represents the hourly mean ± SEM (*n* = 7–8) of NREMS, REMS, and wake. * *p* < 0.05, ** *p* < 0.01, denote significantly different cases from the vehicle (unpaired Student’s *t*-test).

Figure 3 shows the time-course changes in NREMS, REMS, and wake amounts for 12 h after the administration of triphlorethol A (50 mg/kg) and zolpidem (10 mg/kg). Triphlorethol A significantly increased the amount of NREMS during the first 2 h after administration, and this NREMS enhancement was accompanied by a decrease in wake (Figure 3a). There was no further disruption of the sleep architecture during subsequent periods. The significant increase in the amount of NREMS elicited by zolpidem lasted for the first 3 h after administration (Figure 3b). Neither triphlorethol A nor zolpidem had any significant effect on the amount of REMS for 12 h after administration.

### 2.4. Effects of Triphlorethol A on Sleep-Wake Episode Profiles and Delta Activity

To compare the nature of the effects of triphlorethol A on sleep with the hypnotic drug zolpidem, we analyzed the mean duration and total number of NREMS, REMS, and wake episodes. In addition, we calculated delta activity, an indicator of the intensity of NREMS.

**Figure 4:**
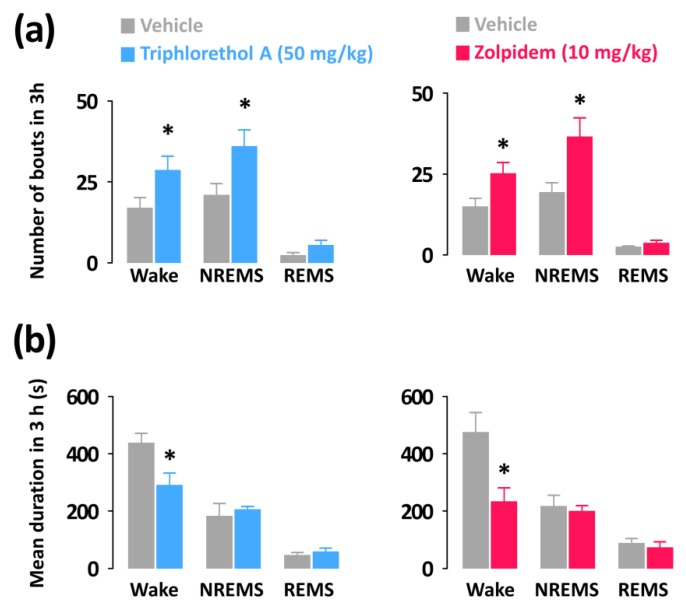
Characteristics of sleep-wake bouts in C57BL/6N mice during the 3 h period after administration of triphlorethol A and zolpidem. (**a**) Changes in the mean duration of wake, NREMS, and REMS bouts. (**b**) Changes in the total number of wake, NREMS, and REMS bouts. Grey bars indicate the baseline day (vehicle). Each value represents the mean ± SEM of each group (*n* = 7–8). * *p* < 0.05 denotes significantly different cases from the vehicle (unpaired Student’s *t*-test).

Both triphlorethol A (50 mg/kg) and zolpidem (10 mg/kg) significantly increased the number of wake (triphlorethol A: 1.69-fold, *p* < 0.05; zolpidem: 1.68-fold, *p* < 0.05) and NREMS (triphlorethol A: 1.71-fold, *p* < 0.05; zolpidem: 1.89-fold, *p* < 0.05) bouts without affecting those of REMS (Figure 4a). In addition, the mean durations of wake bouts were decreased by administration of triphlorethol A and zolpidem (Figure 4b).

**Figure 5:**
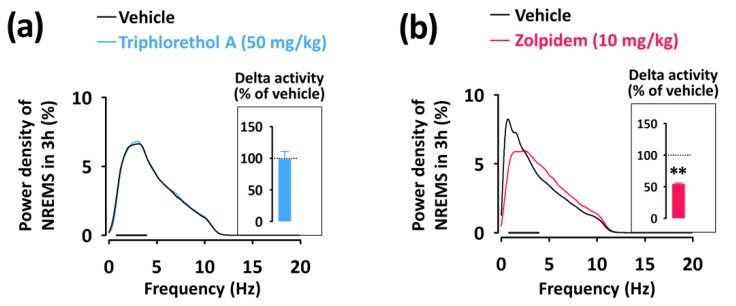
EEG power density curves of NREMS caused by triphlorethol A (**a**) and zolpidem (**b**). Delta activity in NREMS, an index of sleep intensity, is shown in the inset histogram. The bar (—) represents the range of the delta wave (0.5–4 Hz). ** *p* < 0.01, denotes significantly different cases from the vehicle (unpaired Student’s *t*-test).

To evaluate the sleep intensity, we determined the delta activity (the frequency range of 0.5–4 Hz) from the EEG power density during NREMS (Figure 5). Zolpidem significantly (*p* < 0.01) decreased delta activity of NREMS and increased activity in the frequency range of 4.5–11.5 Hz (theta activity) (Figure 5b). However, triphlorethol A did not affect the EEG power density including delta activity in NREMS compared to the vehicle (Figure 5a).

## 3. Discussion

Phlorotannins, which exist within brown seaweeds, are a dietary polyphenol compound. Over the last decade, the biological properties of phlorotannins have been widely investigated, and research on phlorotannins is steadily growing. In our previous studies [11,17], the sleep-promoting effects and GABAergic signaling mechanisms of phlorotannins were demonstrated for the first time. Phlorotannin preparations have been shown to significantly decrease sleep latency and increase NREMS in C57BL/6N mice via the GABA_A_-BZD receptors [11]. The sleep-promoting effects of phlorotannin supplements at 500 mg/kg were comparable to those of the positive control, diazepam at 6 mg/kg [11]. In addition, in a randomized, double-blind, placebo-controlled clinical trial of 20 volunteers [17], phlorotannin supplements significantly decreased wakefulness after sleep onset (phlorotannin vs. placebo, −25.5 ± 30.5 vs. −1.7 ± 14.9; *p* = 0.045) and total wake time (phlorotannin vs. placebo, −0.9 ± 3.0 vs. −6.1 ± 6.8; *p* = 0.048) compared to the placebo.

Triphlorethol A, one of the major phlorotannin constituents, has been reported to exhibit antioxidative effects [18,19,20]. In our previous study [10], triphlorethol A was for the first time characterized as a GABA_A_-BZD receptor ligand, and its binding affinity (*K*_i_) was 4.419 μM. In addition, in a follow-up study, triphlorethol A was demonstrated to potentiate the GABA-induced currents in dorsal raphe neurons in a concentration-dependent manner (data not shown), and the maximal potentiation value (P_max_, %) was 171%. The GABA_A_-BZD receptor is considered an important target for the development of sedative-hypnotic drugs [21,22]. The GABA_A_-BZD receptor ligands with agonistic activity stimulate the ability of GABA to cause membrane hyperpolarization by allowing the influx of chloride anions (Cl^−^) [23]. As a result, inhibitory neurotransmission is augmented, and therefore, these agents possess sedative-hypnotic activity [24,25].

In this study, the main objective was to evaluate the sleep-promoting effects of triphlorethol A based on EEG and EMG in mice. Analyses of sleep architecture and profiles based on EEG and EMG recordings reveal various parameters that are useful in evaluating the sleep-promoting effects of a drug [26]. It has been reported that various hypnotics, including natural compounds and also well-known drugs such as zolpidem enhance sleep quantity, as indicated by an increased duration of NREMS [27,28,29]. In the present study, we found that triphlorethol A (50 mg/kg) significantly decreased sleep latency and increased the amount of NREMS during the first 3 h after administration (Figure 2). The effects of triphlorethol A were comparable to those of zolpidem, a hypnotic drug, at 10 mg/kg. Moreover, the effects of triphlorethol A lasted for the first 2 h after administration and had no further influence on sleep architecture (Figure 3). These results suggest that triphlorethol A induces NREMS without causing adverse effects after sleep induction [29]. Zolpidem can increase the amounts of NREMS by changing the sleep architecture, such as causing changes in bout number [28,30]. We found that, similar to zolpidem, triphlorethol A significantly increased the total number of wake and NREMS bouts and decreased the mean duration of wake bouts (Figure 4). These results clearly indicate that triphlorethol A inhibited the maintenance of wake [31]. Next, we analyzed delta activity during NREMS to evaluate sleep quality. Delta (0.5–4 Hz) activity is a good indicator of the quality or intensity of NREMS [28,32]. It has been reported that zolpidem increases the sleep quantity in NREMS but decreases delta activity [32]. In this study, zolpidem produced a typical decrease in delta activity as expected; however, triphlorethol A induced physiological sleep without a significant change in delta activity (Figure 5). These results suggest that, unlike zolpidem, triphlorethol A increased the quantity of sleep without reducing sleep quality.

## 4. Materials and Methods

### 4.1. Isolation of Triphlorethol A

**Figure 6:**
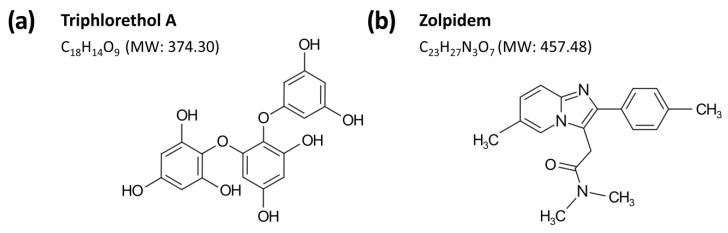
Chemical structures and molecular weights (MW) of triphlorethol A (**a**) and zolpidem (**b**).

Triphlorethol A was isolated from the ethylacetate fraction of edible brown seaweed *Ecklonia cava* ethanol extract using GABA_A_-BZD receptor binding activity-guided fractionation. Dried *E. cava* from Jeju island in Korea was extracted with 80% ethanol at 50 °C for 72 h, and extraction solutions were then filtered and lypophilized. The *E. cava* ethanol extract (98 g) was suspended in H_2_O (1 L) and partitioned with *n*-hexane (10 g), ethylacetate (23 g), and *n*-butanol (11 g) in sequence. The ethylacetate fraction, which showed the most binding activity, was applied to a celite column chromatography (CC) (Ø 15 cm × 15 cm). The column was eluted using a mixture of CHCl_3_-methanol (MeOH) (3:1), and elutes were pooled into 11 sub-fractions (F1–F11) based on thin layer chromatography. The sub-fraction F3 (2.4 g) was further separated with Sephadex LH-20 CC (Sigma-Aldrich, St. Louis, MO, USA) (Ø 2 cm × 50, 80% MeOH) to get 19 sub-fractions (FF1–FF19). Triphlorethol A (Figure 6a) was isolated as FF3 (17 mg) and was identified by ^1^H-NMR and ^13^C-NMR data. Its structural elucidations are summarized as follows: yellow amorphous powder (MeOH); FAB/MS *m*/*z* 373 [M − 1]^−^; ^1^H-NMR (400 MHz, CD_3_OD, δ_H_) 6.05 (1H, d, *J* = 2.8 Hz, H-5), 6.00 (2H, d, *J* = 2.0 Hz, H-2′′,6′′), 5.92 (1H, d, *J* = 2.0 Hz, H-4′′), 5.89 (2H, s, H-3′,5′), 5.74 (1H, d, *J* = 2.8 Hz, H-3); ^13^C-NMR (100 MHz, CD_3_OD, δ_C_) 162.37 (C-1′′), 160.29 (C-3′′,5′′), 156.39 (C-4′), 156.14 (C-4), 153.71 (C-6), 152.56 (C-2), 152.07 (C-2′,6′), 125.65 (C-1), 124.62 (C-1′), 98.01 (C-3), 97.46 (C-4′′), 96.14 (C-3′,5′), 95.37 (C-2′′,6′′), 94.98 (C-5).

### 4.2. Drugs

Zolpidem (Figure 6b) was used as a reference hypnotic drug and obtained from the Ministry of Food and Drug Safety of Korea (Cheongwon-gun, Chungcheongbuk-do, Korea). Pentobarbital was purchased from Hanlim Pharm. Co. Ltd. (Seoul, Korea).

### 4.3. Animals

All procedures involving animals were conducted in accordance with the guidelines of the Korea Food Research Institutional Animal Care and Use Committee (permission number: KFRI-M-09118). Imprinting control region (ICR; male, 18–22 g) and C57BL/6N (male 27–30 g) mice were purchased from Koatech Animal Inc. (Pyeongtaek, Korea). All animals were housed in an insulated, sound-proof recording room maintained at an ambient temperature of 23 ± 0.5 °C, with a constant relative humidity (55 ± 2%) on an automatically controlled 12-h light/dark cycle (lights off at 17:00). Mice had free access to food and water. All efforts were made to minimize animal suffering and the number of animals required for the production of reliable scientific data.

### 4.4. Pentobarbital-Induced Sleep Test

**Figure 7:**
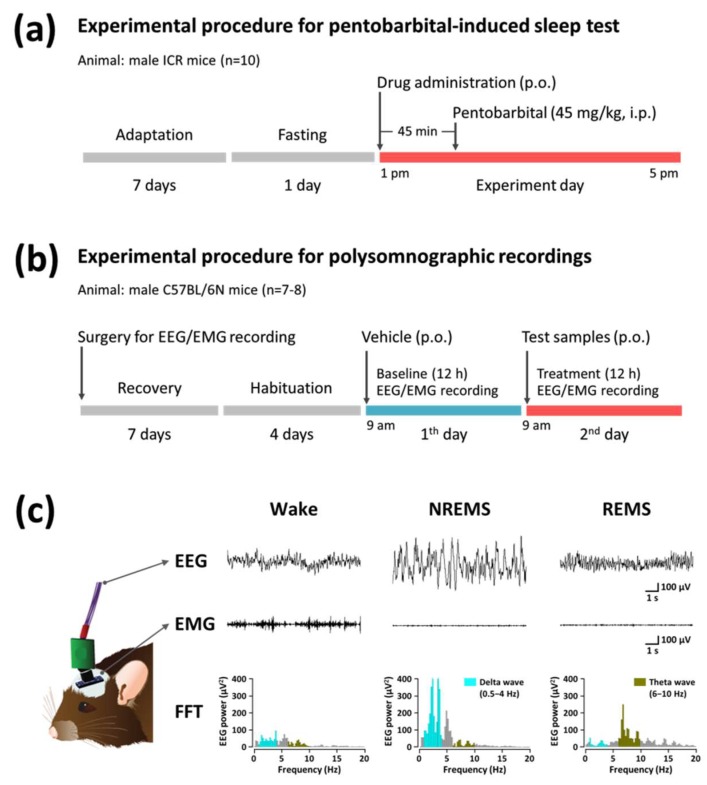
Experimental procedures and timelines for the pentobarbital-induced sleep test (**a**) and polysomnographic recordings (**b**). (**c**) Typical EEG and EMG waveforms, and fast Fourier transform (FFT) spectrum in mice.

The experimental procedures and timeline for the pentobarbital-induced sleep test are shown in Figure 7a. All experiments were performed between 1:00 p.m. and 5:00 p.m., and mice were fasted for 24 h before experiment onset. Mice (*n* = 10) received drugs (p.o.) 45 min before pentobarbital injection. Control mice (vehicle solution: 0.5% carboxymethyl cellulose, 10 mL/kg) were tested in parallel with animals receiving drug treatments. After the administration of pentobarbital (45 mg/kg, i.p.), mice were placed in individual cages and observed for measurements of sleep latency and duration. Sleep latency was recorded from the time of pentobarbital injection to the time of sleep onset, and sleep duration was defined as the difference in time between the loss and recovery of the righting reflex. Observers who scored these variables were blind to the treatment groups.

### 4.5. Polygraphic Recordings and Vigilance State Analysis

The experimental procedures and timeline for polygraphic recordings and sleep analysis are shown in Figure 7b. Under pentobarbital anesthesia (50 mg/kg, i.p.), C57BL/6N mice were chronically implanted with a head mount (#8201, Pinnacle Technology Inc., Lawrence, KS, USA) installed with EEG and EMG electrodes for polysomnographic recordings. The front edge of the head mount was placed 3.0 mm anterior to bregma, and four electrode screws for EEG recording were positioned in holes perforated into the skull. Two EMG wire electrodes were inserted into the nuchal muscles. The head mount was fixed to the skull with dental cement. After surgery, mice were allowed to recover in individual cages for seven days and habituated to the recording conditions for four days before the experiment.

The EEG and EMG recordings were carried out by means of a slip ring designed so that the movement of the mice was not restricted. EEG and EMG were recorded using the PAL-8200 data acquisition system (Pinnacle Technology Inc., Lawrence, KS, USA). The EEG and EMG signals were amplified (100×), filtered (low-pass filter: 25 Hz EEG and 100 Hz EMG), and stored at a sampling rate of 200 Hz. Sleep states were monitored for a period of 12 h, on both baseline and experimental days. Baseline recordings were taken for each animal for 12 h, beginning at 09:00. These baseline recordings served as controls for the same animal. All drugs were dissolved in sterile saline containing 5% Tween 80 immediately before use, and orally administered (p.o.) to mice.

The mice were considered asleep when they showed no EMG signal. The vigilance states were automatically classified by a 10 s epoch as wakefulness (wake), rapid eye movement sleep (REMS), or non-REM sleep (NREMS) by SleepSign ver. 3.0 (Kissei Comtec, Nagano, Japan). As a final step, defined sleep-wake stages were examined visually and corrected if necessary. The sleep latency was defined as the time from drug administration to the appearance of the first NREMS episode lasting for at least 120 s. Bouts of NREMS, REMS, and wake were defined as periods of one or more consecutive epochs (each epoch: 10 s). The delta power of NREMS in the range of 0.5–4 Hz was first summed and then normalized as a percentage of the corresponding mean delta power of NREMS. Representative EEG/EMG waveforms and fast Fourier transform (FFT) spectrum of delta and theta waves are shown in Figure 7c.

### 4.6. Statistical Analysis

All data are expressed as the mean ± SEM. Statistical analysis was performed with Prism 5.0 (GraphPad Software Inc., San Diego, CA, USA). For multiple comparisons, data were analyzed using a one-way analysis of variance (ANOVA) followed by Dunnett’s test. Comparisons between two groups of data were analyzed using an unpaired Student’s *t*-test. The significance level was set at *p* < 0.05 for all statistical tests.

## 5. Conclusions

In summary, triphlorethol A (50 mg/kg) promoted NREMS and decreased sleep latency in mice, and its effects were comparable to those of the well-known hypnotic drug zolpidem (10 mg/kg). Unlike zolpidem, triphlorethol A induced NREMS similar to physiological NREMS without a significant decrease in delta activity, an index of sleep intensity. Our demonstration of the sleep-promoting effects of triphlorethol A provides important evidence supporting the sleep-promoting effects of phlorotannins. Moreover, we propose that triphlorethol A may be a promising structure for developing novel sedative hypnotics. To better understand the sleep-promoting effects and the precise mechanisms by which triphlorethol A acts, further studies are needed to investigate its effects on different subtypes of the GABA_A_ receptor.
